# Sensitivity Analysis of Hepatitis B and C Mortality in England Using Data Linkage: Meeting the WHO Elimination Threshold for Mortality

**DOI:** 10.1111/jvh.70016

**Published:** 2025-03-26

**Authors:** Annabel A. Powell, Annastella Costella, Rachel Roche, David Leeman, Ashley Brown, Beatrice Emmanouil, Mark Gillyon‐Powell, Ross Harris, Holly D. Mitchell, Ruth Simmons, Monica Desai

**Affiliations:** ^1^ Blood Safety, Hepatitis, Sexually Transmitted Infections (STIs) and HIV Division UK Health Security Agency London UK; ^2^ Department of Hepatology Imperial College London, Imperial College NIHR BRC London UK; ^3^ HCV Elimination NHS England London UK; ^4^ Blizard Institute, Faculty of Medicine and Dentistry Queen Mary University of London London UK

**Keywords:** data linkage, hepatitis B, hepatitis C, mortality

## Abstract

The United Kingdom, along with many other countries, is working towards eliminating viral hepatitis as a public health threat by 2030, with a combined mortality target of less than or equal to six deaths per 100,000 population. The current methodology of reporting uses death registrations alone, which has been estimated to underestimate mortality rates by up to 60% for hepatitis C (HCV)‐related liver disease. We aim to conduct a sensitivity analysis using data linkage of death certificates, hepatitis B (HBV) and HCV diagnoses and admissions for end‐stage liver disease (ESLD) and/or hepatocellular carcinoma (HCC) to estimate mortality rates, assess progress towards elimination and evaluate underreporting. Between 2000 and 2023, 7967 deaths were reported due to HBV‐ and/or HCV‐associated ESLD and/or HCC. Using data linkage of all three datasets, this increased to 11,487, with underreporting estimated to be 37% overall. The upper bound combined mortality rate was estimated to be 1.3 deaths per 100,000 population at its peak in 2015, therefore surpassing the WHO target for all years evaluated. From 2015 to 2023, both HCV‐associated ESLD and/or HCC mortality decreased (1.12 to 0.88 deaths per 100,000 population), however, there was a slight increase for HBV‐associated ESLD and/or HCC deaths during the same time frame (0.3 to 0.35). A higher proportion of HBV‐related deaths were in males (*p* < 0.05) who died outside London (*p* < 0.05) and a lower proportion were White (*p* < 0.05) when compared to HCV‐related deaths. While England has met the WHO impact targets, it is important we continue to drive reductions in mortality.

## 1 Introduction

The burden of liver disease, while being largely preventable, is increasing in England compared to a decline in most other European countries, with key risk factors being alcohol use, obesity and viral hepatitis [1]. People living with chronic hepatitis C (HCV) or B (HBV) are at an increased risk of liver cirrhosis, end‐stage liver disease (ESLD) and hepatocellular carcinoma (HCC), which cause severe disease and death.

Many countries, including the United Kingdom, are working towards the WHO global strategy of eliminating viral hepatitis as a public health threat by 2030, with a mortality target of less than or equal to six deaths per 100,000 population. This previously included a target of two deaths per 100,000 population due to HCV and four deaths per 100,000 population due to HBV. It was reported in England in 2023 that the annual mortality rate was 0.44 per 100,000 population due to HCV and 0.15 for HBV; therefore, the target has been surpassed using the current reporting methodology [2, 3].

Considerable progress has been made in reducing HCV‐related ESLD/HCC death registrations over the last 10 years within England, with a 34.0% reduction in HCV‐related death registrations between 2012 and 2022 [2], largely driven by access to curative direct‐acting antivirals, which have shorter treatment durations, are better tolerated and are more effective than previous interferon‐based regimens. The same gains, however, have not been seen for HBV, with HBV‐related ESLD and/or HCC mortality remaining stable between 0.13 and 0.19 deaths per 100,000 population since 2005; for 2022, this was estimated to be 0.15 [3]. Internationally, in low‐ and middle‐income countries from 2019 to 2022, HCV‐related deaths have declined; however, an increase in HBV‐related deaths has been seen [4], largely attributed to an ageing population cohort and COVID‐19–related disruptions to treatment access.

Death registrations are routinely used to calculate mortality rates; however, we have previously noted that underreporting of HCV as a cause of death could be as much as 44%–60% for liver disease‐related causes [5], a limitation of using death registrations alone. This is consistent with estimations from the United States, which found that 41% of persons with HCV dying of liver‐related causes had HCV mentioned on the death certificate, as well as other countries, including Switzerland and Scotland [6, 7, 8]. Using data linkage between the Office for National Statistics (ONS) Death Registry in England, Hospital Episode Statistics (HES), and routine laboratory reports of hepatitis diagnoses to enhance HBV and/or HCV diagnosis, we aim to conduct a sensitivity analysis to evaluate the accuracy of estimated mortality rates, assess progress towards meeting the WHO target for mortality and evaluate underreporting of viral hepatitis‐related deaths.

## 2 Methods

### 2.1 Data Sources

#### 2.1.1 Routine Laboratory Reports of Hepatitis Diagnoses—Second‐Generation Surveillance System (SGSS)

HCV and/or HBV diagnoses were obtained from routine laboratory reports of HCV or HBV, defined as the detection of HCV antibody (anti‐HCV) or HCV RNA in blood for HCV and the detection of hepatitis B surface antigen (HBsAg) for HBV, submitted by virology laboratories in England to UKHSA. The laboratory reporting system does not distinguish between anti‐HCV and HCV RNA in an individual, and so laboratory ‘confirmed’ cases are a mix of current (viraemic) and ever‐infected individuals. Laboratory reports of hepatitis have been submitted to UKHSA (previously Public Health England) through surveillance forms or electronically since 1990; laboratory reporting became mandatory in 2010. Reports include basic demographics (name, date of birth, sex and NHS number) and variable risk factor information. Through linkage with the Patient Demographic Service, reported information can be improved, particularly for persons reported to UKHSA with an NHS number and date of birth; sex, date of death, patient address and registered General Practice (GP) can be updated. Name, date of birth, sex and NHS number are used to de‐duplicate reports.

#### 2.1.2 Office for National Statistics (ONS) Cause of Death Registry

The registry of deaths at ONS includes all reported deaths in England since 1937. Cause of death is coded according to the tenth revision of the International Statistical Classification of Diseases and Related Health Problems (ICD10) as the underlying cause or as one of nine contributory causes. Cause of death groupings used is the same as previously reported (Table 1) [9]. Identifiers available for linkage include NHS number, date of birth, patient address and full name. Date of death is used to calculate the year of death.

**TABLE 1:** Cause of death groupings, with ICD10 codes.

Cause of death grouping	ICD10 codes
Viral hepatitis	B15‐19
End‐stage liver disease (ESLD)	I850, I983, K704, K720, K721, K729, K767, R18
Hepatocellular carcinoma	C220

#### 2.1.3 Hospital Episode Statistics

Hospital episode statistics contain patient and clinical information for all inpatient (including day cases), outpatient and accident and emergency episodes. Inpatient stays and day cases (hospital admissions) have been collected since 1989 and have been used for the analysis. Diagnoses associated with admission are coded using ICD‐10. Outpatient and accident and emergency data were excluded as diagnosis codes are not collected within the accident and emergency dataset and are poorly completed in the outpatient dataset. Identifiers available for linkage include NHS number, sex, date of birth and patient address.

#### 2.1.4 Data Linkage

Routine laboratory reports of hepatitis and ONS data were linked using a stepwise deterministic approach; that is, the data were first linked using NHS number in conjunction with date of birth (then dropping either the day, month or year), the data were then linked with NHS number, soundex (a phonetic algorithm for indexing names) and initial before finally being linked with NHS number, soundex and year of birth. In instances where the NHS number was not available, we matched on soundex, initial and date of birth. A hierarchical approach was used for each year where matches by NHS number superseded a match without an NHS number. Additional checks were conducted on records where a person had been tested after their date of death, where a person has been linked to a death recorded in a previous year and where multiple records matched.

HES and ONS linkage is routinely conducted by NHS Digital using NHS Number or linking on date of birth, sex and postcode. Detailed methodology can be found here: Linkage methodology—NHS England Digital.

HES and SGSS data were linked using a two‐step process, with persons first linked to all HES inpatient records using NHS number or hospital number; matches are verified using additional variables such as date of birth, sex and postcode.

**FIGURE 1:**
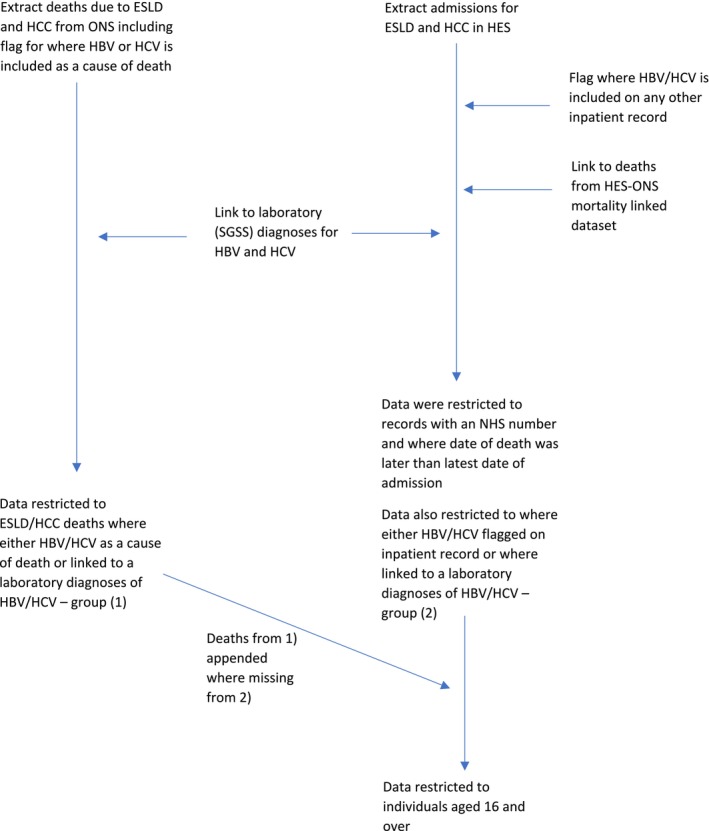
Methodology flow diagram to extract and link cohort.

HES inpatient records for ESLD and HCC were extracted and linked to HES inpatient records where HBV and/or HCV were included on any inpatient record using HES identifiers (Figure 1).

Ethnicity data are supplemented through data linkage between HES and SGSS.

#### 2.1.5 Outcome Definitions

Liver‐related mortality (ESLD or HCC) associated with viral hepatitis (B/C) was identified using six different scenarios (Table 2), producing an upper and lower bound. Unless otherwise specified, causes of death are either underlying or contributory. This was analysed for HBV and HCV individually and combined. Scenario 1a (lower bound): death registrations of ESLD and/or HCC with HBV and/or HCV reported on the death certificate; Scenario 1b: Scenario 1a and/or linked to a routine laboratory report of HBV and/or HCV; Scenario 2a: Inpatient record of ESLD and/or HCC with HBV and/or HCV reported as a contributing factor linked to death registrations (HES‐ONS linked mortality); Scenario 2b: scenario 2a and/or with HBV and/or HCV reported on the death certificate; Scenario 2c: scenario 2b and/or linked to a routine laboratory report of HBV and/or HCV; and Scenario 2c + 1b (upper bound): Scenario 2c and Scenario 1b. Data were presented as the number of deaths over time and mortality rates shown per 100,000 population.

**TABLE 2:** Source of hepatitis diagnosis.

	Source of viral hepatitis diagnosis			
	ONS deaths	Hospital admission	Laboratory reports	HES‐linked mortality
Scenario 1a	X			
Scenario 1b	X		X	
Scenario 2a		X		
Scenario 2b		X		X
Scenario 2c		X	X	X
Scenario 2c + 1b	X	X	X	X

Data were managed in SQL and analysed in RStudio and Microsoft Excel. P‐values shown were calculated using the chi‐squared test.

#### 2.1.6 Ethics

This work is covered by Health Service (Control of Patient Information) Regulation 2002 (regulations 3 and 7) which makes provisions for the recognition, control and prevention of communicable diseases and other risks to public health. Therefore, ethical approval from participants was not needed, but appropriate Caldicott approval was obtained for linkage between databases.

## 3 Results

**FIGURE 2:**
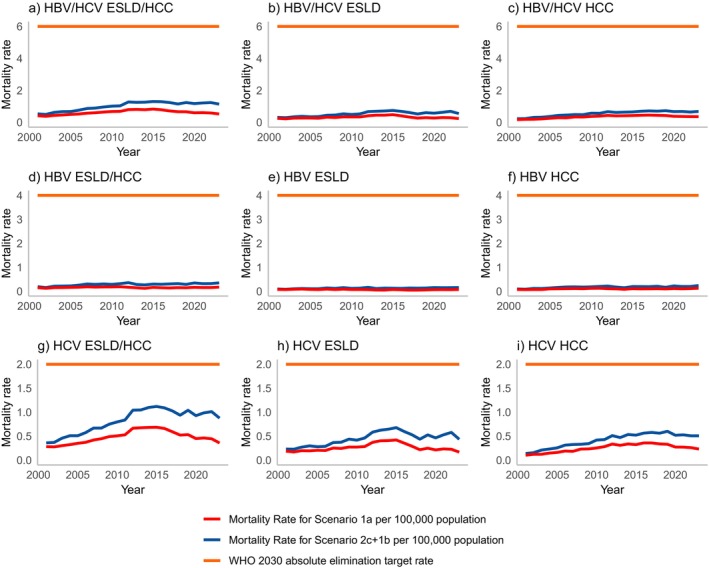
Sensitivity analyses of the number of deaths due to ESLD and/or HCC that are attributable to HBV and/or HCV over time. The mortality rate for the upper bound (Scenario 2c + 1b) is shown to compare with the WHO absolute elimination target rate.

*Scenario 1a*: (Figure 2) Between 2001 and 2023, 134,787 individuals were reported to ONS death registrations to have died with a cause of death reported as ESLD and/or HCC. Of these individuals, 7967 had viral hepatitis reported on the death certificate (Table 4); HBV was reported for 2005 and HCV for 6198.

*Scenario 1b*: Linking ONS death registrations to routine laboratory reports for viral hepatitis, the total number of individuals who died of ESLD and/or HCC with a record of viral hepatitis increased by 3520 to 11,487. This includes 2627 and 9271 individuals for HBV and HCV respectively.

*Scenario 2a*: During the same time frame, 738,922 individuals had an inpatient record for ESLD (718,601) and/or HCC (46,254). Of these individuals, 29,278 had viral hepatitis recorded on their inpatient record, 8263 with HBV and 23,300 with HCV. Of the 29,278 individuals with viral hepatitis on their inpatient record, 8865 were linked to a death registration of ESLD and/or HCC (scenario 2a).

*Scenario 2b*: This increased to 9776, where an inpatient record for ESLD and/or HCC was linked to a death registration where HBV and/or HCV was recorded on the death certificate, including 2775 and 7859, respectively, for HBV and/or HCV (Scenario 2b).

*Scenario 2c*: Linking HES inpatient records to routine laboratory reports for HBV and HCV increases the number of deaths to 10,780, including 3004 for HBV and 8741 for HCV (Scenario 2c).

*Scenario 2c + 1b*: Overlaying Scenarios 2c (10,780) and 1b (11,487), the highest estimated number of ESLD or HCC deaths attributable to HCV and/or HBV was 12,590 (Table 4), including 3497 for HBV and 10,128 for HCV.

### 3.1 Crude Mortality Rates

Crude mortality rates for HBV and/or HCV using the upper bound decreased from 1.30 in 2015 to 1.14 in 2023 (Figure 2 and Tables 3). Crude mortality rates for the lower bound, Scenario 1a, decreased from 0.87 in 2015 to 0.54 in 2023.

**TABLE 3:** Upper (2c + 1b) and lower (1a) bounds of mortality rates per 100,000 population for HBV/HCV‐related ESLD/HCC.

	HBV and/or HCV						HBV						HCV					
	ESLD and/or HCC		ESLD		HCC		ESLD and/or HCC		ESLD		HCC		ESLD and/or HCC		ESLD		HCC	
Year of Death	Upper	Lower	Upper	Lower	Upper	Lower	Upper	Lower	Upper	Lower	Upper	Lower	Upper	Lower	Upper	Lower	Upper	Lower
2001	0.53	0.42	0.31	0.25	0.23	0.17	0.19	0.15	0.1	0.08	0.1	0.07	0.36	0.29	0.23	0.19	0.14	0.11
2002	0.5	0.4	0.29	0.24	0.23	0.19	0.15	0.13	0.08	0.07	0.09	0.07	0.37	0.29	0.23	0.18	0.16	0.13
2003	0.62	0.45	0.35	0.28	0.31	0.2	0.21	0.15	0.1	0.09	0.12	0.07	0.46	0.32	0.27	0.21	0.21	0.13
2004	0.67	0.47	0.38	0.27	0.32	0.22	0.22	0.16	0.11	0.1	0.12	0.07	0.51	0.33	0.3	0.2	0.23	0.15
2005	0.68	0.52	0.35	0.28	0.37	0.26	0.22	0.17	0.11	0.08	0.14	0.11	0.51	0.37	0.28	0.22	0.26	0.16
2006	0.76	0.55	0.36	0.27	0.44	0.3	0.26	0.17	0.11	0.07	0.16	0.11	0.57	0.39	0.29	0.21	0.31	0.2
2007	0.87	0.59	0.44	0.33	0.46	0.29	0.3	0.19	0.14	0.09	0.18	0.11	0.67	0.43	0.37	0.26	0.33	0.19
2008	0.89	0.64	0.46	0.33	0.49	0.35	0.29	0.18	0.12	0.08	0.19	0.12	0.67	0.47	0.37	0.27	0.33	0.24
2009	0.96	0.67	0.53	0.36	0.48	0.34	0.3	0.18	0.15	0.09	0.18	0.11	0.75	0.51	0.44	0.29	0.34	0.25
2010	1.01	0.71	0.49	0.36	0.57	0.39	0.29	0.2	0.12	0.08	0.19	0.13	0.8	0.53	0.42	0.29	0.42	0.27
2011	1.03	0.72	0.53	0.35	0.57	0.41	0.32	0.2	0.13	0.08	0.2	0.13	0.84	0.55	0.46	0.29	0.43	0.3
2012	1.27	0.83	0.67	0.43	0.67	0.45	0.36	0.17	0.16	0.07	0.22	0.11	1.04	0.7	0.59	0.39	0.51	0.35
2013	1.25	0.84	0.69	0.47	0.62	0.42	0.28	0.15	0.12	0.05	0.18	0.1	1.05	0.71	0.63	0.42	0.47	0.32
2014	1.26	0.83	0.71	0.47	0.64	0.43	0.27	0.13	0.13	0.05	0.15	0.09	1.1	0.71	0.65	0.43	0.54	0.35
2015	1.3	0.87	0.74	0.51	0.65	0.44	0.3	0.17	0.13	0.07	0.2	0.11	1.12	0.72	0.68	0.44	0.52	0.34
2016	1.29	0.82	0.69	0.43	0.69	0.46	0.29	0.15	0.12	0.07	0.2	0.1	1.09	0.69	0.6	0.38	0.56	0.37
2017	1.24	0.75	0.62	0.34	0.72	0.48	0.31	0.14	0.13	0.05	0.19	0.11	1.03	0.63	0.53	0.3	0.58	0.39
2018	1.15	0.69	0.52	0.27	0.7	0.46	0.32	0.15	0.13	0.05	0.21	0.11	0.94	0.55	0.44	0.23	0.56	0.36
2019	1.24	0.69	0.61	0.3	0.73	0.45	0.29	0.15	0.14	0.06	0.17	0.1	1.04	0.55	0.53	0.26	0.6	0.35
2020	1.17	0.62	0.57	0.28	0.67	0.39	0.35	0.17	0.15	0.07	0.22	0.11	0.93	0.47	0.47	0.22	0.52	0.28
2021	1.21	0.62	0.63	0.31	0.68	0.37	0.32	0.15	0.15	0.07	0.2	0.1	0.99	0.48	0.53	0.24	0.53	0.28
2022	1.24	0.61	0.69	0.3	0.65	0.37	0.32	0.16	0.15	0.07	0.2	0.11	1.01	0.47	0.58	0.24	0.51	0.27
2023	1.14	0.54	0.55	0.24	0.68	0.37	0.35	0.18	0.16	0.08	0.24	0.13	0.88	0.37	0.44	0.17	0.51	0.24

**TABLE 4:** Numbers of deaths per year for the upper (2c + 1b) and lower (1a) bounds of HBV/HCV‐related ESLD/HCC.

	HBV and/or HCV						HBV						HCV					
	ESLD and/or HCC		ESLD		HCC		ESLD and/or HCC		ESLD		HCC		ESLD and/or HCC		ESLD		HCC	
Year of Death	Upper	Lower	Upper	Lower	Upper	Lower	Upper	Lower	Upper	Lower	Upper	Lower	Upper	Lower	Upper	Lower	Upper	Lower
2001	262	208	154	126	113	86	95	75	47	38	48	37	179	142	115	94	69	52
2002	246	199	144	118	116	95	77	64	41	37	43	34	184	146	114	91	78	63
2003	312	224	174	138	155	99	106	77	52	44	59	36	229	158	136	103	105	65
2004	336	234	190	138	162	109	109	79	57	48	58	36	256	164	150	98	117	75
2005	343	262	177	144	187	134	113	87	54	41	69	54	258	185	142	111	130	83
2006	388	278	184	139	222	154	131	89	54	37	82	56	293	198	147	108	160	101
2007	446	304	228	170	236	151	156	98	71	48	93	57	344	223	189	133	168	100
2008	463	330	238	169	252	180	149	94	63	39	96	61	347	246	194	138	172	123
2009	502	348	277	186	251	180	159	96	79	47	92	57	392	266	229	149	180	128
2010	534	373	260	188	302	205	152	103	64	44	98	67	420	281	220	151	221	142
2011	548	381	280	187	301	217	168	104	69	41	107	69	445	293	246	156	228	157
2012	682	444	359	229	356	241	193	93	88	38	116	60	558	372	313	207	274	188
2013	675	453	373	252	334	226	152	79	62	29	95	54	564	380	337	227	254	174
2014	686	450	387	255	347	236	145	71	72	28	80	48	597	387	351	233	291	191
2015	714	474	408	277	356	240	164	91	70	41	108	59	614	393	372	242	286	185
2016	714	455	380	239	383	253	161	84	67	36	108	56	603	384	331	209	312	206
2017	690	419	343	189	399	269	170	79	75	27	107	59	572	349	295	167	322	215
2018	643	386	292	153	392	256	178	84	73	28	117	60	525	307	245	126	315	200
2019	698	387	342	171	411	252	161	85	76	34	94	57	586	312	297	145	339	197
2020	664	350	325	160	380	220	196	94	87	38	126	64	525	265	263	127	294	160
2021	686	351	355	174	384	212	179	87	84	39	112	58	558	269	299	138	299	157
2022	707	349	393	174	369	212	182	90	86	39	114	65	578	267	331	138	290	154
2023	651	308	315	137	390	211	201	102	89	43	136	75	501	211	249	97	290	138

Crude mortality rates for ESLD attributable to HBV and HCV using the upper bound decreased from 0.74 in 2015 to 0.55 per 100,000 in 2023 (Figure 2, Tables 3). This reduction is driven by deaths attributable to HCV (0.68 in 2015 to 0.44 in 2023), with an increase for HBV (0.13 in 2015 to 0.16 in 2023). Crude mortality rates for HCC attributable to HBV and HCV per 100,000 population peaked in 2019 at 0.73, falling to 0.68 per 100,000 in 2023. Crude mortality rates for HCC attributable to HBV have remained consistent over time, rising slightly from 0.20 in 2015 to 0.24 in 2023, whereas mortality rates for HCC attributable to HCV have decreased following a peak in 2019 of 0.60 to 0.51 in 2023.

### 3.2 Demographics

Of the 12,590 individuals who died of ESLD and/or HCC attributable to HBV/HCV, 76.6% were male, the median age at death was 59 years (IQR: 51–67) and, where ethnicity was reported (90.1%), 68.5% of individuals were of White ethnicity (Table 5). Seventy per cent of those who had died were reported to have died outside of London, and HBV/HCV coinfection was identified in 1035 individuals, with a higher proportion of those with HBV coinfected compared to HCV (30% vs. 10%). Demographics differed between individuals who died attributable to HCV compared with HBV. There was a higher proportion of males among those with HBV compared to HCV (80.9% vs. 75.5% respectively; *p* < 0.05), and a higher proportion of those with HBV died outside of London when compared to HCV (72.4% vs. 62.3%, respectively, *p* < 0.05), whereas a lower proportion with HBV was of White ethnicity compared to those with HCV (76.0% vs. 47.4% respectively p < 0.05). For those with HBV, 21.9% were of Asian ethnicity and 13.1% were of Black ethnicity compared to 9.8% and 4.1% respectively for HCV. There was little difference in median age at death for those with HBV and HCV at 59 years (IQR: 50–69) versus 58 years (IQR: 51–66), respectively.

**TABLE 5:** Demographics.

	Overall		Hepatitis B		Hepatitis C	
	*n*	%	*n*	%	*n*	%
Total	12,590		3497		10,128	
Age group						
18–29	94	0.7	54	1.5	49	0.5
30–49	2880	22.9	880	25.2	2259	22.3
50–69	7267	57.7	1789	51.2	6075	60.0
70+	2336	18.6	767	21.9	1737	17.2
Unknown	13	0.1	7	0.2	8	0.1
Sex						
Male	9639	76.6	2828	80.9	7648	75.5
Female	2950	23.4	668	19.1	2480	24.5
Unknown	1	0.0	1	0.0		
Ethnicity						
Asian	1624	12.9	765	21.9	997	9.8
Black	799	6.3	459	13.1	416	4.1
Mixed	141	1.1	63	1.8	91	0.9
Other	151	1.2	77	2.2	93	0.9
White	8630	68.5	1657	47.4	7695	76.0
Unknown	1245	9.9	476	13.6	836	8.3
Region						
London	3142	25.0	1161	33.2	2282	22.5
Outside London	8816	70.0	2178	62.3	7335	72.4
Unknown	632	5.0	158	4.5	511	5.0
Coinfection	1035	8.2	1035	29.6	1035	10.2

## 4 Discussion

Using laboratory reports of hepatitis surveillance, alongside inpatient and mortality records, we estimate the upper and lower bounds of ESLD and HCC mortality attributable to HBV and/or HCV in England, with the highest mortality rate of 1.30 deaths per 100,000 population in 2015 well below the WHO impact target of a combined mortality rate of ≤ 6/100,000 per year for HCV and HBV. The mortality rates from 2015 to 2023 for ESLD ranged from 0.74 to 0.55 and for HCC from 0.65 to 0.68, with the highest estimates for both ESLD and HCC similarly below the 2030 WHO combined mortality target for HBV and HCV of 6 deaths per 100,000 population, below the HBV‐related mortality rate of four deaths per 100,000 population per year and the HCV‐related mortality rate of two deaths per 100,000 population per year.

Both ESLD and HCC mortality associated with HCV have decreased since the introduction of the hepatitis C elimination programme, which has included the roll‐out of curative direct‐acting antivirals and case‐finding initiatives to (re)engage individuals in the care pathway. While both HCV‐related ESLD and HCC mortality have decreased, the decrease for ESLD was much earlier than that observed for HCC. This difference is likely to be associated with treatment in persons with advanced disease, with a greater impact on regression of fibrosis and improved liver function in those with cirrhosis, rather than removing the risk of liver cancer. This highlights the importance of early diagnosis and early treatment, which are essential if further declines are to be achieved. Several countries—Australia, the United States, Spain and Scotland have—also reported decreases in mortality associated with HCV, highlighting the positive impact the DAAs, coupled with a scale‐up in case finding and testing activities, have had [10, 11, 12, 13]. Decreases reported range from 30% to 70% and, in most cases, the observed decrease follows a peak in mortality in the early 2000s [10, 11, 12, 13, 14].

Our data are consistent with the United States, which reported an age‐adjusted rate of 0.42 HBV‐related deaths and a plateauing in recent years; they, however, have seen a decline since 2013 [15]. Australia and Canada also presented decreases in HBV‐related mortality [10, 16] attributable to increased vaccination, treatment, enhanced HCC screening and improved management of patients, unlike what has been observed in England. The data presented for HBV showed a fluctuation in deaths due to both ESLD and/or HCC associated with HBV over time. For ESLD, this increases from 0.12 in 2010 to 0.16 in 2023; for HCC, this is 0.19 to 0.24 for 2010 and 2023, respectively. Treatment for HBV is not curative, and historically, treatment eligibility in the United Kingdom has been complex and requires regular patient follow‐up, often with no indication for treatment; therefore, attrition in retention in care can be seen [3]. The WHO has recently simplified guidance on HBV treatment, which may make it easier for patients to understand the benefits of care and stay engaged [17]. They have also lowered the threshold of treatment eligibility, which may increase the number of patients indicated for treatment sooner, who may be more likely to remain in care compared to those monitored with no indication for treatment [3, 17]. Another reason for this may be attributed to an ageing population, particularly among those living with chronic HBV with no current curative therapies [10]. The Global Burden of Disease study found that developed countries, including the United States and the United Kingdom, had both an increase in the number of deaths and mortality rate between 2000 and 2019, as well as an ageing population, with the proportion aged 65 and over increasing from 16% to 19% during that time frame [18]. Furthermore, there is a high estimated undiagnosed fraction for HBV compared to HCV, estimated at 53%, which will exacerbate disease progression if an individual remains undiagnosed for longer, leading to a higher burden of severe disease and death [3]. A global modelling study estimates 14% diagnosed globally, ranging from 6% overall in low‐income countries to 45% in high‐income countries [19]. Additionally, in England, a universal programme was introduced in 2017, and before that, a selective programme; however, the majority of cases are among migrants from endemic countries [20]. As a result, vaccination programmes in these endemic countries may have a greater impact on reducing HBV prevalence in England than the domestic programme. These heterogeneous populations affected by HBV in England also experience a disproportionate burden of disease, with poorer access and outcomes to healthcare. Barriers to accessing testing and treatment include language, stigma, sociocultural factors and limited understanding of HBV infection and its harms [21, 22, 23, 24, 25]. Similar efforts for HBV are needed to mirror the extensive work already undertaken to increase testing and treatment coverage for hepatitis C; however, this is currently not possible without political prioritisation and financial commitment.

The demographics of those who died largely reflect that of the diagnosed population, with a higher proportion of males and a higher proportion of individuals of White ethnicity for those with HCV, whereas those with HBV who died were more likely to be of Asian or Black ethnicity. It is important to highlight that even though we report a decrease in ESLD and/or HCC mortality attributable to HCV, the median age for those dying still suggests that individuals are dying prematurely (aged 30–69 years) and adds further evidence to the health inequalities experienced by these populations. High rates of HBV/HCV coinfection were identified in our cohort, with 8% of all deaths identified with a coinfection, which was higher in patients with HBV (30%) compared to HCV (10%). HBV/HCV coinfection has been shown to result in higher rates of cirrhosis, HCC and death; [16, 26] however, there are few detailed studies on the proportion of deaths caused by HBV/HCV coinfection, and many analyses also include HIV coinfection. Data from Spain, Italy, Japan, Taiwan and Iran show approximately 10%–15% of patients with chronic HBV infection are infected with HCV, whereas 2%–10% of anti‐HCV–positive patients were HBsAg positive [27]. These data are slightly lower than the proportion with coinfection in our analysis; however, as we are presenting deaths and with a higher expected mortality rate among those coinfected, this may explain the difference. Further work will be required to better characterise individuals identified with HBV/HCV coinfection in England, as well as better understand their diagnosis and management.

Underreporting of viral hepatitis on a person's death certificate, comparing the upper bound with reporting on death certificates only, was 37% overall, 43% for HBV and 39% for HCV. Underreporting of viral hepatitis remains an issue in mortality records, which makes it difficult to collate data to validate mortality reductions. To address this gap, we used data linkage from several established healthcare datasets. In countries where these datasets may not be available, the WHO has suggested using the attributable fraction of HCC as well as decompensated cirrhosis due to HBV and HCV from clinic‐based sentinel sites over the mortality envelope. Work is underway to pilot estimating the attributable fraction in sentinel sites across England and align these with the mortality rates calculated using surveillance data.

A limitation of using established healthcare datasets is that linkage between datasets relies on sufficient patient identifiers; therefore, we may be underestimating rates as these records were excluded. The expectation, however, is that many of these individuals may be duplicated in the datasets; therefore, reducing this underestimation. Additionally, it is not possible to differentiate between current and past infections among those with HCV. Our methodology is also not able to distinguish the underlying cause of ESLD and/or HCC; therefore, our presented mortality rates may be an overestimation as we indicate the presence of a viral hepatitis diagnosis. Only inpatient data were used, although many patients with ESLD and/or HCC are managed through either outpatient or emergency care. This may be mitigated as our outcome is death; many of the patients, if not admitted to hospital at any point for ESLD and/or HCC or if it is listed as a cause of death, will be captured using Scenario 1a and 1b.

## 5 Conclusion

Through data linkage, we estimate the upper and lower bounds of ESLD and HCC mortality attributable to HBV and/or HCV in England, with the highest estimate falling below the WHO threshold for mortality from viral hepatitis B and C of ≤ 6/100,000 per year, one of the impact targets for validation of elimination. This was also observed for HCV‐related mortality ≤ 2/100,000 per year and HBV mortality ≤ 4/100,000 per year. While England has met the WHO impact targets, it is important we continue to drive reductions in mortality. In particular, the later decrease in HCC mortality compared with ESLD highlights the importance of early diagnosis and access to treatment.

## Conflicts of Interest

The authors declare no conflicts of interest.

## Data Availability

The data that support the findings of this study are available on request from the corresponding author. The data are not publicly available due to privacy or ethical restrictions.

## References

[1] Department for Health and Social Care , “Liver Disease Profiles,” (2024), accessed October 10, 2024, https://fingertips.phe.org.uk/profile/liver‐disease#0.

[2] UK Health Security Agency , “Hepatitis C in England 2023,” (2024), accessed October 10, 2024, https://www.gov.uk/government/publications/hepatitis‐c‐in‐the‐uk/hepatitis‐c‐in‐england‐2023#:~:text=In%20England%2C%20around%2062%2C600%20adults,in%202015%20(Figure%201).

[3] UK Health Security Agency , Hepatitis B in England 2024 (UK Health Security Agency, 2024).

[4] World Health Organisation , Global Hepatitis Report 2024 (World Health Organisation, 2024).

[5] R. Simmons , G. Ireland , S. Ijaz , M. Ramsay , and S. Mandal , “Causes of Death Among Persons Diagnosed With Hepatitis C Infection in the Pre‐ and Post‐DAA Era in England: A Record Linkage Study,” Journal of Viral Hepatitis 26, no. 7 (2019): 873–880. 30896055 10.1111/jvh.13096

[6] O. Keiser , F. Giudici , B. Müllhaupt , et al., “Trends in Hepatitis C‐Related Mortality in Switzerland,” Journal of Viral Hepatitis 25, no. 2 (2018): 152–160. 29159841 10.1111/jvh.12803

[7] R. Mahajan , J. Xing , S. J. Liu , et al., “Mortality Among Persons in Care With Hepatitis C Virus Infection: The Chronic Hepatitis Cohort Study (CHeCS), 2006–2010,” Clinical Infectious Diseases 58, no. 8 (2014): 1055–1061. 24523214 10.1093/cid/ciu077

[8] S. A. McDonald , S. J. Hutchinson , S. M. Bird , et al., “The Growing Contribution of Hepatitis C Virus Infection to Liver‐Related Mortality in Scotland,” Euro Surveillance 15, no. 18 (2010): 2110. 20460092

[9] G. Ireland , S. Mandal , M. Hickman , M. Ramsay , R. Harris , and R. Simmons , “Mortality Rates Among Individuals Diagnosed With Hepatitis C Virus (HCV); an Observational Cohort Study, England, 2008 to 2016,” Eurosurveillance 24, no. 30 (2019): 1800695. 31362807 10.2807/1560-7917.ES.2019.24.30.1800695 PMC6668288

[10] S. Tillakeratne , S.‐A. Pearson , M. Alavi , et al., “Trends in Viral Hepatitis Liver‐Related Morbidity and Mortality in New South Wales, Australia,” Lancet Regional Health – Western Pacific 51 (2024): 8051. 10.1016/j.lanwpc.2024.101185 PMC11402402 39282135

[11] J. Politi , J. M. Guerras , M. Donat , et al., “Favorable Impact in Hepatitis C‐Related Mortality Following Free Access to Direct‐Acting Antivirals in Spain,” Hepatology 75, no. 5 (2022): 1247–1256. 34773281 10.1002/hep.32237

[12] S. A. McDonald , S. T. Barclay , H. A. Innes , et al., “Uptake of Interferon‐Free DAA Therapy Among HCV‐Infected Decompensated Cirrhosis Patients and Evidence for Decreased Mortality,” Journal of Viral Hepatitis 28, no. 9 (2021): 1246–1255. 34002914 10.1111/jvh.13543

[13] F. Carrat , H. Fontaine , C. Dorival , et al., “Clinical Outcomes in Patients With Chronic Hepatitis C After Direct‐Acting Antiviral Treatment: A Prospective Cohort Study,” Lancet 393, no. 10179 (2019): 1453–1464. 30765123 10.1016/S0140-6736(18)32111-1

[14] Y. Sahakyan , V. Lee‐Kim , K. E. Bremner , J. M. Bielecki , and M. D. Krahn , “Impact of Direct‐Acting Antiviral Regimens on Mortality and Morbidity Outcomes in Patients With Chronic Hepatitis c: Systematic Review and Meta‐Analysis,” Journal of Viral Hepatitis 28, no. 5 (2021): 739–754. 33556225 10.1111/jvh.13482

[15] Prevention CfDCa , “National Viral Hepatitis Progress Report 2024,” (2024), accessed December 12, 2024, https://www.cdc.gov/hepatitis/policy/npr/2024/NationalProgressReport‐HepB‐ReduceDeaths.htm.

[16] J. D. Makuza , D. Jeong , S. Wong , et al., “Association of Hepatitis B Virus Treatment With All‐Cause and Liver‐Related Mortality Among Individuals With HBV and Cirrhosis: A Population‐Based Cohort Study,” Lancet Regional Health – Americas 36 (2024): 36. 10.1016/j.lana.2024.100826 PMC11261267 39040565

[17] World Health Organisation , “Guidelines for the Prevention, Diagnosis, Care and Treatment for People With Chronic Hepatitis B Infection,” (2024), accessed December 12, 2024, https://www.who.int/publications/i/item/9789240090903. 10.1097/ID9.0000000000000128 PMC11462912 39391287

[18] C. Zhang , Y. Liu , H. Zhao , and G. Wang , “Global Patterns and Trends in Total Burden of Hepatitis B From 1990 to 2019 and Predictions to 2030,” Clinical Epidemiology 14 (2022): 1519–1533. 36540899 10.2147/CLEP.S389853 PMC9760077

[19] D. Razavi‐Shearer , I. Gamkrelidze , C. Pan , et al., “Global Prevalence, Cascade of Care, and Prophylaxis Coverage of Hepatitis B in 2022: A Modelling Study,” Lancet Gastroenterology & Hepatology 8, no. 10 (2023): 879–907. 37517414 10.1016/S2468-1253(23)00197-8

[20] S. Hahné , M. Ramsay , K. Balogun , W. J. Edmunds , and P. Mortimer , “Incidence and Routes of Transmission of Hepatitis B Virus in England and Wales, 1995‐2000: Implications for Immunisation Policy,” Journal of Clinical Virology 29, no. 4 (2004): 211–220. 15018847 10.1016/j.jcv.2003.09.016

[21] A. Cochrane , P. Collins , and J. P. Horwood , “Barriers and Opportunities for Hepatitis B Testing and Contact Tracing in a UK Somali Population: A Qualitative Study,” European Journal of Public Health 26, no. 3 (2016): 389–395. 26896472 10.1093/eurpub/ckv236

[22] A. Cochrane , I. Evlampidou , C. Irish , S. M. Ingle , and M. Hickman , “Hepatitis B Infection Prevalence by Country of Birth in Migrant Populations in a Large UK City,” Journal of Clinical Virology 68 (2015): 79–82. 26071342 10.1016/j.jcv.2015.05.009

[23] M. Guirgis , F. Nusair , Y. M. Bu , K. Yan , and A. T. Zekry , “Barriers Faced by Migrants in Accessing Healthcare for Viral Hepatitis Infection,” Internal Medicine Journal 42, no. 5 (2012): 491–496. 22151101 10.1111/j.1445-5994.2011.02647.x

[24] K. Hacker , M. Anies , B. L. Folb , and L. Zallman , “Barriers to Health Care for Undocumented Immigrants: A Literature Review,” Risk Management Healthc Policy 8 (2015): 175–183. 10.2147/RMHP.S70173 PMC4634824 26586971

[25] B. Rechel , P. Mladovsky , D. Ingleby , J. P. Mackenbach , and M. McKee , “Migration and Health in an Increasingly Diverse Europe,” Lancet 381, no. 9873 (2013): 1235–1245. 23541058 10.1016/S0140-6736(12)62086-8

[26] R. L. Kruse , J. R. Kramer , G. L. Tyson , et al., “Clinical Outcomes of Hepatitis B Virus Coinfection in a United States Cohort of Hepatitis C Virus‐Infected Patients,” Hepatology 60, no. 6 (2014): 1871–1878. 25065513 10.1002/hep.27337 PMC4245372

[27] D. Konstantinou and M. Deutsch , “The Spectrum of HBV/HCV Coinfection: Epidemiology, Clinical Characteristics, Viral Interactions and Management,” Annals of Gastroenterology 28, no. 2 (2015): 221–228. 25830779 PMC4367211

